# Supplementary material for: Machine Learning Prediction Models for Mortality in Intensive Care Unit Patients with Lactic Acidosis
Source: J Clin Med. 2021 Oct 28;10(21):5021. doi: 10.3390/jcm10215021 (PMC8584535; doi:10.3390/jcm10215021)
Supplement: Supplementary file 1 [file jcm-10-05021-s001.zip › jcm-1415826-sup.pdf]

Figure S1. Correlation of variables in the dataset

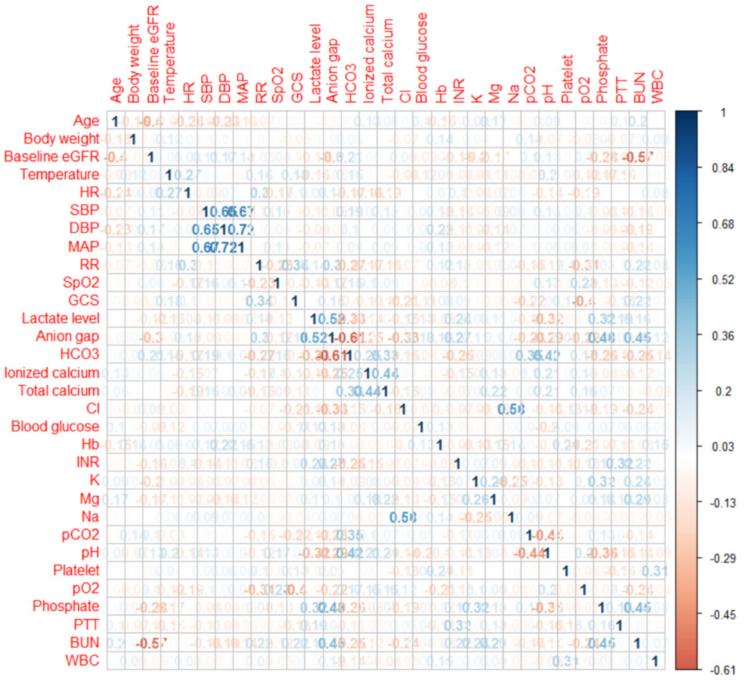

Figure S2. Pruned DT based on cross-validated error results using the complexity parameter associated with the minimal error

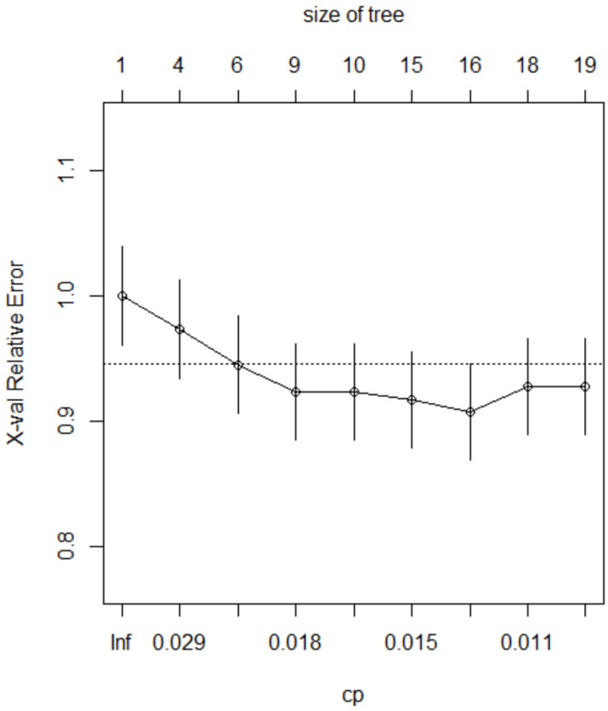

Figure S3. Error rate of RF model by number of trees

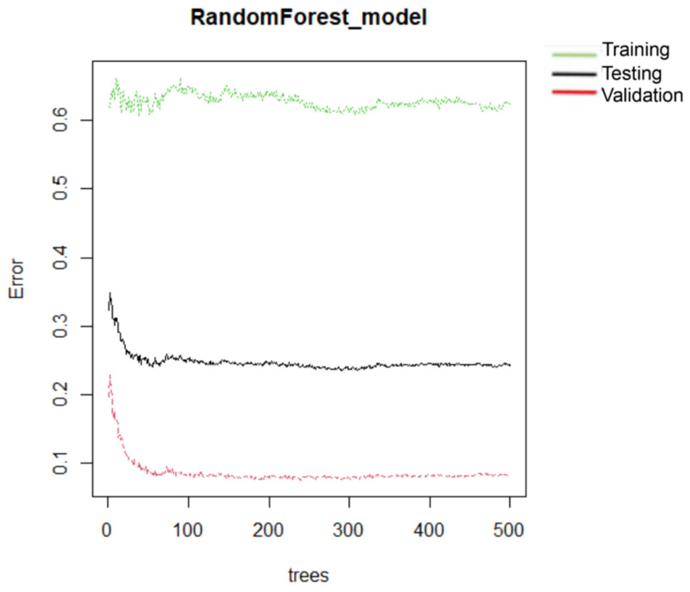

Figure S4. Calibration plot of RF model

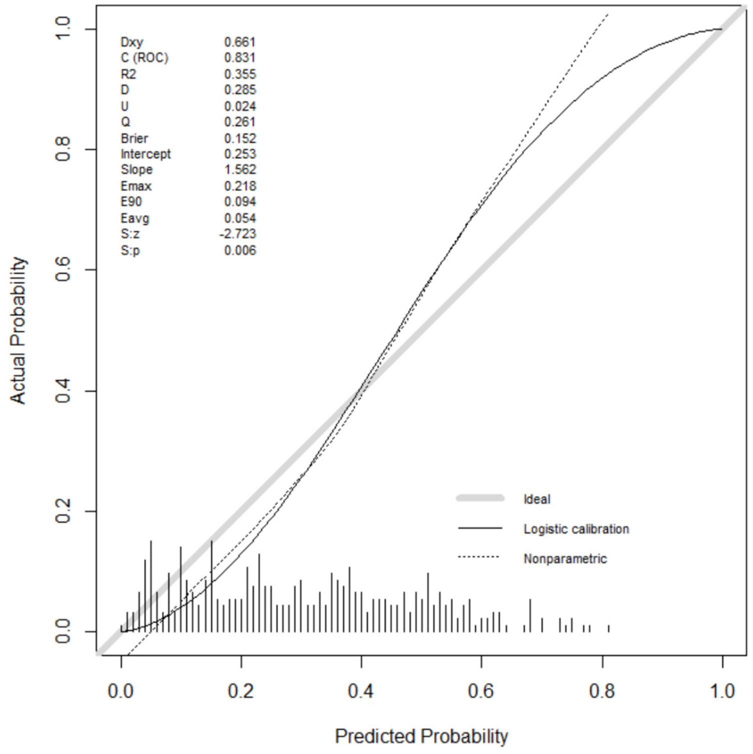

Figure S5 Calibration plot of Decision Tree model

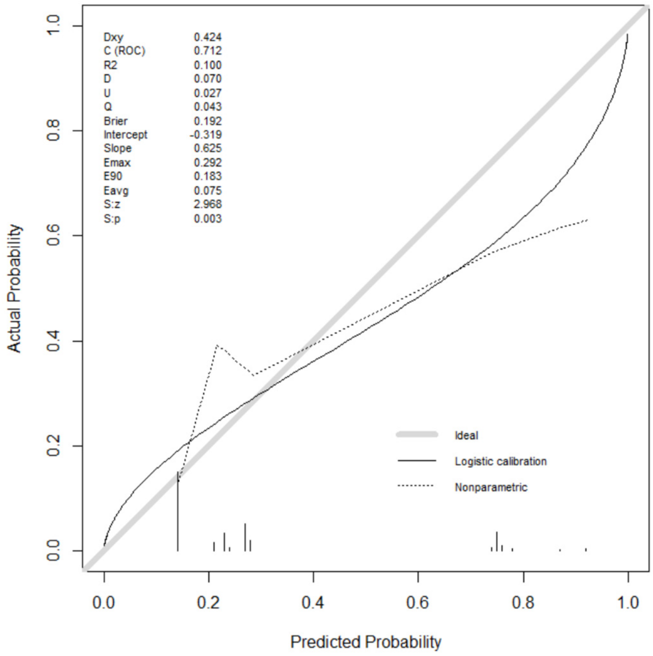

Figure S6 Calibration plot of Xgboost model

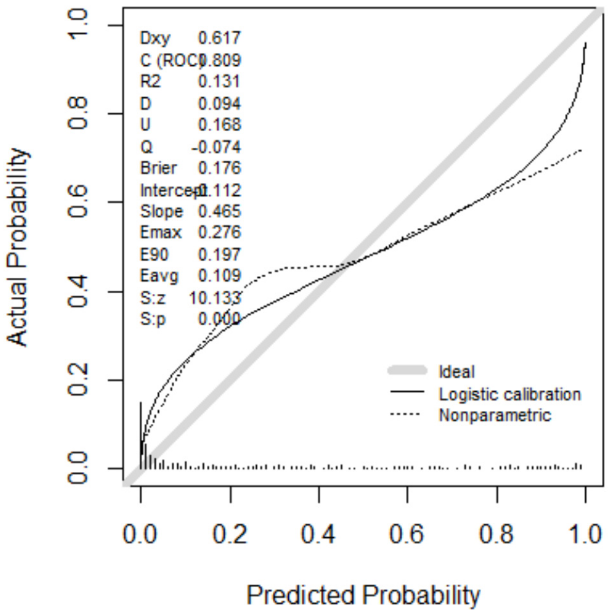

Figure S7 Calibration plot of ANN model

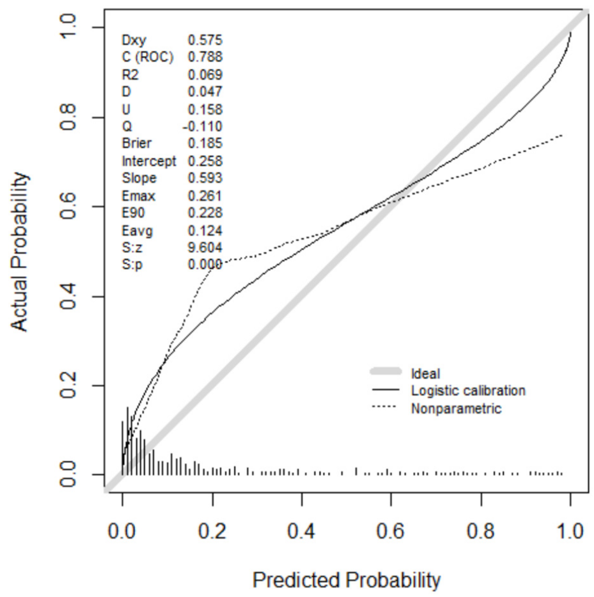

Figure S8 Calibration plot of LR model

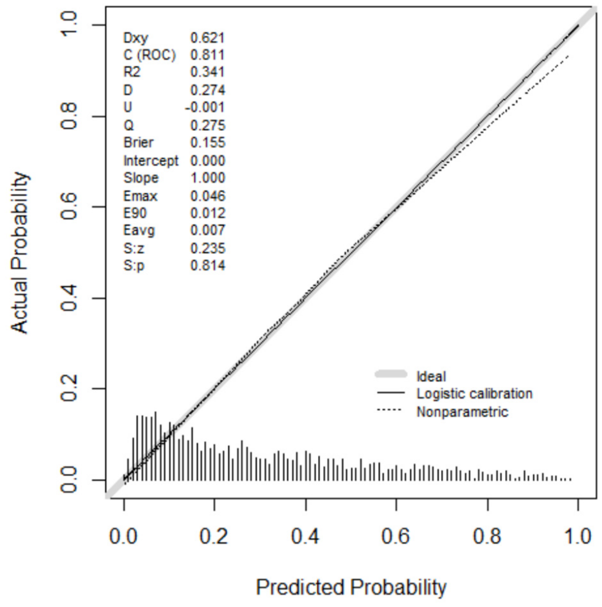

Figure S9 Calibration plot of SAPS II score

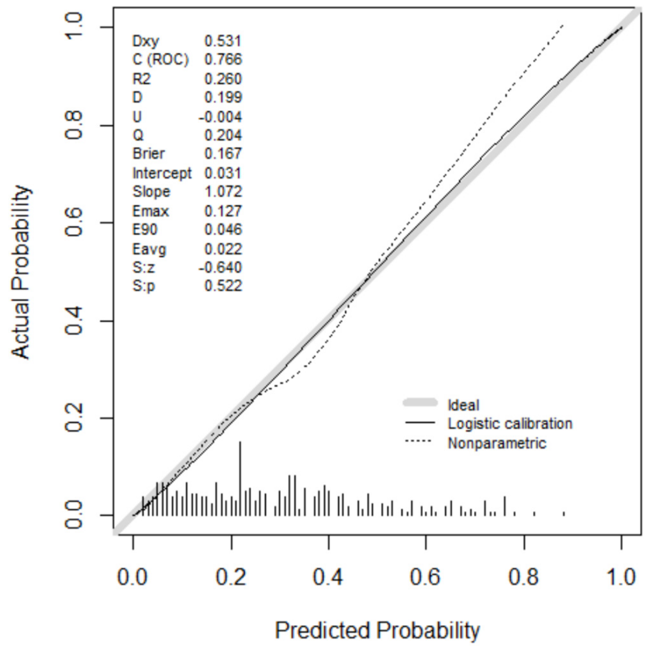

Figure S10 Calibration plot of Charlson score

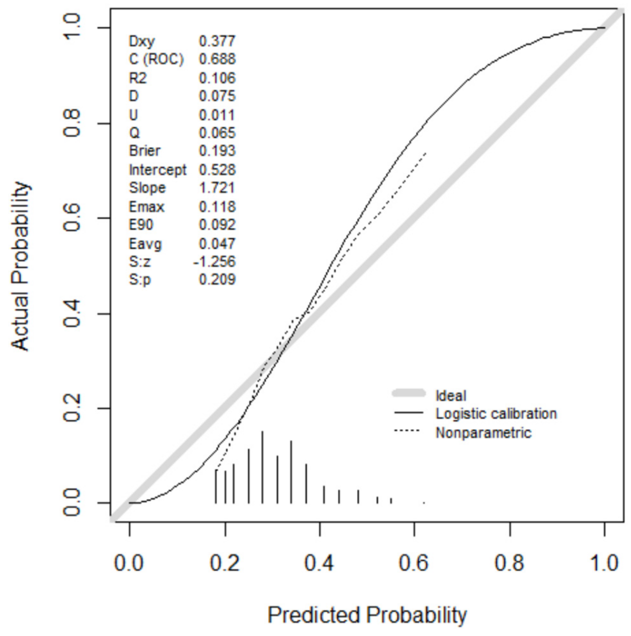

Supplementary method

Decision Tree

For DT analysis, the number of terminal nodes was determined considering the scree plot showing the relationship between the tree size and coefficient of variance. The decision tree was pruned based on cross-validated error results using the complexity parameter associated with the minimal error.

```
library(rpart)
library(rpart.plot)
classifier = rpart(formula = hospital.death ~ .,
data = training_set, method = "class")
plotcp(classifier)
min_cp = classifier$cptable[which.min(classifier$cptable[,"xerror"]),"CP"]
Rpart_prune = prune(classifier, cp = min_cp)
prp(Rpart_prune)
prp(Rpart_prune, type = 1)
rpart.plot(Rpart_prune)
classifier1 = rpart(formula = hospital.death ~ .,
data = training_set_scaled, method = "class")
plotcp(classifier1)
min_cp1 = classifier1$cptable[which.min(classifier1$cptable[,"xerror"]),"CP"]
Rpart_prune1 = prune(classifier1, cp = min_cp)
```

Random Forest

```
randomForest(formula = hospital.death ~ ., data = training_set_scaled, importance = TRUE)
Type of random forest: classification
Number of trees: 500
No. of variables tried at each split: 8
```

XGBoost

```
library(caret)
xgb_trcontrol = trainControl(
method = "cv",
number = 5,
allowParallel = TRUE,
verboseIter = FALSE,
returnData = FALSE
)
xgbGrid<-expand.grid(nrounds = c(100,200),
max_depth = c(10,15,20,25),
colsample_bytree = seq(0.5, 0.9, length.out = 5),
eta = c(0.1, 0.2, 0.3),
gamma = c(0, 0.1, 0.2, 0.3),
min_child_weight = 1,
subsample = 1)
set.seed(123)
xgb_model = train(hospital.death~.,
```

```
data = training_set_onehot,
trControl = xgb_trcontrol,
tuneGrid = xgbGrid,
method = "xgbTree")
xgb_model$finalModel
```

ANN

Grid Hyper-Parameter Search

```
hyper_params <- list(
activation=c("Rectifier","Tanh","Maxout","RectifierWithDropout","TanhWithDropout","MaxoutWithDropout"),
hidden=list(c(20,20),c(50,50),c(30,30,30),c(25,25,25,25)),
input_dropout_ratio=c(0,0.05),
l1=seq(0,1e-4,1e-6),
l2=seq(0,1e-4,1e-6)
)
hyper_params
response = "hospital.death"
predictors = setdiff(names(training_set_scaled), response)
df = as.h2o(training_set_scaled)
dim(df)
df
splits <- h2o.splitFrame(df, c(0.6,0.2), seed=1234)
train <- h2o.assign(splits[[1]], "train.hex") # 60%
valid <- h2o.assign(splits[[2]], "valid.hex") # 20%
test <- h2o.assign(splits[[3]], "test.hex") # 20%
## Stop once the top 5 models are within 1% of each other
search_criteria = list(strategy = "RandomDiscrete", max_runtime_secs = 360, max_models = 100, seed=1234567,
stopping_rounds=5, stopping_tolerance=1e-2)
dl_random_grid <- h2o.grid(
algorithm="deeplearning",
grid_id = "dl_grid_random",
training_frame=train,
validation_frame=valid, #we need validation set
x=predictors,
y=response,
epochs=1,
stopping_metric="logloss",
stopping_tolerance=1e-2, ## stop when logloss does not improve by >=1% for 2 scoring events
stopping_rounds=2,
score_validation_samples=10000, ## downsample validation set for faster scoring
score_duty_cycle=0.025, ## don't score more than 2.5% of the wall time
max_w2=10, ## can help improve stability for Rectifier
hyper_params = hyper_params,
search_criteria = search_criteria
)
grid <- h2o.getGrid("dl_grid_random",sort_by="logloss",decreasing=FALSE)
grid
grid@summary_table[1,]
best_model <- h2o.getModel(grid@model_ids[[1]]) ## model with lowest logloss
best_model
```

Grid ID: dl_grid_random

Used hyper parameters:

- activation
- hidden
- input_dropout_ratio
- l1
- l2

Number of models: 200

Number of failed models: 0

Hyper-Parameter Search Summary: ordered by increasing logloss

activation hidden model_ids logloss

1 Rectifier [50, 50] dl_grid_random_model_66 0.4994253167872157

2 Rectifier [30, 30, 30] dl_grid_random_model_199 0.5089895341734378

3 Tanh [25, 25, 25, 25] dl_grid_random_model_98 0.510901171105774

4 MaxoutWithDropout [30, 30, 30] dl_grid_random_model_164 0.5111197239857912

5 Tanh [25, 25, 25, 25] dl_grid_random_model_172 0.5116825184286653

---

activation hidden model_ids logloss

195 MaxoutWithDropout [20, 20] dl_grid_random_model_26 0.7386979547364945

196 Maxout [50, 50] dl_grid_random_model_111 0.7523729924895673

197 Maxout [25, 25, 25, 25] dl_grid_random_model_158 0.7604032308880321

198 MaxoutWithDropout [30, 30, 30] dl_grid_random_model_64 0.8740278285670754

199 MaxoutWithDropout [25, 25, 25, 25] dl_grid_random_model_88 0.896520552510747

200 MaxoutWithDropout [25, 25, 25, 25] dl_grid_random_model_156 0.9319084051367906

```
ANNBestmodel = h2o.deeplearning(y = 'hospital.death',
training_frame = as.h2o(training_set_scaled),
activation = 'Rectifier',
hidden = c(50,50),
epochs = 1,
seed = 1234751,
input_dropout_ratio = 0.05,
l1 = 4.5e-05,
l2 = 3e-06,
max_w2 = 10,
distribution = 'bernoulli',
score_validation_samples = 10000,
score_duty_cycle = 0.025,
stopping_rounds = 2,
stopping_metric = 'logloss',
stopping_tolerance = 0.01,
max_runtime_secs = 350.293,
train_samples_per_iteration = -2)
```

Evaluation indices

In assessing the classification efficiency of all classifiers, a confusion matrix is important. It is a 2-2 matrix that offers details about the real and forecast classifications. There are four components in the confusion matrix: true positive (TP), true negative (TN), FP, and FN. A patient who dies can be classified correctly (TP) or incorrectly (FN), and a patient who survives can be classified correctly (TN) or incorrectly (FP).

The evaluation indices are defined as:

- Accuracy = (TP + TN)/(TP + TN + FP + FN).
- Precision = TP/(TP + FP)
- ERR = (FP+ FN)/(TP+TN+FN+FP)
- MCC = ((TP x TN)-(FP x FN))/sqrt(((TP + FP)(TP + FN)(TN + FP)(TN + FN)))
- F-score = (2 x precision x recall)/(precision + recall).
